# Molecular and in situ characterization of cadmium-resistant diversified extremophilic strains of *Pseudomonas* for their bioremediation potential

**DOI:** 10.1007/s13205-013-0155-z

**Published:** 2013-07-18

**Authors:** Sourabh Jain, Arun Bhatt

**Affiliations:** 1 Uttarakhand Technical University, Dehradun, India; 2 National Research Centre on Plant Biotechnology, IARI Campus, Pusa, New Delhi India; 3 Department of Crop Improvement, Uttarakhand University of Horticulture and Forestry, Uttarakhand, India

**Keywords:** AAS, Psychrotolerant, Alkaliphile, Bioremediation, Cadmium

## Abstract

Cadmium-resistant strains psychrotolerant *Pseudomonas putida* SB32 and alkalophilic *Pseudomonas monteilli* SB35 were originally isolated from the soil of Semera mines, Palamau, Jharkhand, India. Further, to unravel the mechanism involved in cadmium resistance, plasmid DNA was isolated from the strains and subjected to amplification of the *czc* gene, which is responsible for the efflux of three metal cations, viz. Co, Zn and Cd, from the cell. Furthermore, the amplicon was cloned into pDrive cloning vector and sequenced. When compared with the available database, the sequence homology of the cloned gene showed the presence of a partial *czcA* gene sequence, thereby indicating the presence of a plasmid-mediated efflux mechanism for resistance in both strains. These results were further confirmed by atomic absorption spectroscopy and transmission electron microscopy. Moreover, the strains were characterized functionally for their bioremediation potential in cadmium-contaminated soil by performing an in situ experiment using soybean plant. A marked increase in agronomical parameters was observed in presence of both strains. Further, the concentration of metal ions decreased in both plants and soil in the presence of these bioinoculants.

## Introduction

Naturally occurring bacteria that are capable of metal accumulation have been extensively studied since it is difficult to imagine that a single bacterium would remove all heavy metals from its polluted site (Clausen 2000). Therefore, diversified microorganisms have to be classified morphologically, physiologically and biochemically to understand their taxonomical variation and evolutionary distance from the parental linage.

Cadmium is a non-redox metal unable to produce active oxygen species (AOS) via Fenton and IQ Haber–Weiss reaction (Sanita Di Toppi and Gabbrielli 1999). However, several reports demonstrate that Cd can indirectly promote the generation of AOS (Sandalio et al. 2001). Cadmium-increased lipid peroxidation has been demonstrated in *Phaseolus vulgaris* roots and leaves (Chaoui et al. 1997). When accumulated in the plant tissue, it causes alteration in catalytic efficacy of enzymes (Somashekaraiah et al. 1992; Romero-Puertas et al. 1999; Piqueras et al. 1999), damage to the cellular membranes (Tu and Brovillett 1987) and inhibits the root growth. This metal enters the environment mainly from fertilizers and is transferred to animals and humans through industrial processes (Wagner 1993) leading to serious damage to human health (Alloway 1990). It affects cell proliferation, differentiation, apoptosis and increases oncogene activation to carcinogenesis (Naidu et al. 1997).

Microbial survival in polluted soil depends on intrinsic biochemical and structural properties, physiological and/or genetic adaptations including morphological changes in cells and environmental modifications of metal speciation (Wuertz and Mergeay 1997). Microbes apply various types of resistance mechanisms in response to heavy metals (Nies 2003). Microbial methods of environment purification and cleanup are known to be the most promising because of their safety, efficiency, and cost-effectiveness (He et al. 2011). Many microorganisms can absorb and concentrate heavy metals thus, providing resistance (Burke and Pfister 1986). On the other hand, studying metal ion resistances give us important insights into environmental processes and provide an understanding of basic living processes. Furthermore, genes for resistance to inorganic salts of soft metals are found both on plasmids and in chromosomes. The physiological role of plasmid-encoded determinants is generally to confer resistance; however, mostly chromosomally encoded systems may include metal ion homeostasis (Wuertz and Mergeay 1997).

Two well-studied genetic mechanisms of metal resistance in bacteria include heavy metal efflux systems (Nies and Silver 1995) and the presence of metal binding proteins (Robinson et al. 1990). Many operons of efflux system are known. In the Gram-positive bacteria, the plasmid-encoded Cd efflux system, called the CadA resistance system, utilizes the CadA protein, which is a P-type ATPase (Tsai and Linet 1993). However, Cd resistance in Gram-negative organisms is due to a multi-protein chemiosmotic antiport system (Silver 1996). Primarily, the *czc* system detoxifies the cell by cation efflux, the three metal cations, viz. cobalt, zinc and cadmium, which are taken up into the cell by fast and unspecific transport system for Mg^2+^ are actively extruded from cell by products of *czc* resistance determinants (Nies et al. 1989b). The protein complex is composed of three subunits, *czc*C, *czc*B and *czc*A (Nies et al. 1989a). *Czc*A is a RND protein (Tseng et al. 1999) and contains two hydrophilic domains, which are located in the periplasm (Goldberg et al. 1999), as the other two subunits of *czc* complex, *czc*B and *czc*C. There are at least six regulatory proteins (*Czc*D, *Czc*R, *Czc*S, *Czc*N, *Czc*I and an unknown sigma factor, “RpoX”) involved in regulation of the three structural genes *czcCBA*. The proteins *czc*A and *czc*B alone are capable of pumping zinc ions out of the cell, even if *czc*C is not present (Nies et al. 1989b). Therefore, *czc*A is proposed to function as the actual efflux-transportation protein; however, *czc*A’s specificity for exportation of these ions is apparently regulated by the presence of two other structural proteins (Nies and Silver 1995). The cell surfaces of all microorganisms are negatively charged due to the presence of various anionic structures which gives bacteria the ability to bind metal cations. So, isolation of heavy metal-resistant bacteria may be useful to improve the application of microorganism in environment protection.

In this study, an effort has been made to unravel the mechanism involved in Cd resistance in psychrotolerant *P. putida* SB32 and alkalophilic *P. monteilli* SB35. This includes molecular characterization of the strains to identify the location of *czc* gene and to find out whether the resistance to Cd was due to efflux mechanism or due to accumulation of metal in the bacterial cell. Furthermore, in situ trials on soybean plant in presence of the two isolates were performed to analyze the bioremediation potential of strains.

## Materials and methods

### Isolation of Cd-resistant extremophilic isolates

Psychrotolerants *P. putida* SB32 and alkalophilic *P. monteilli* SB35 strains were isolated from the soil sample of Semera Mines, Palamau, Jharkhand, India. The strains were grown in nutrient broth (Himedia Laboratories Private Limited, Mumbai, India) containing 1 mM cadmium chloride (CdCl_2_) and was kept overnight at 30 °C. Furthermore, the grown culture was also streaked on nutrient agar plates (Himedia Laboratories) and the plates were kept at 4 °C until further use. They were optimized for their respective parameters, i.e., temperature for psychrotolerant *P. putida* SB32 (optimum growth at 20 °C) and pH for alkalophilic *P. monteilli* SB35 (pH optima 9.0) and are found to be possessing high resistance to Cd.

### Plasmid and genomic DNA isolation

The plasmid DNA was isolated using previously described protocol (Anderson and McKay 1983) and genomic DNA by Qiagen Bacterial genomic DNA isolation kit, Hilden, Germany. The isolated DNA was made to run on 0.8 % agarose gel and visualized subsequently.

### Amplification of czc gene

The *czc* gene was amplified from both plasmid and genomic DNA of strains SB32 and SB35 using primers *czc*F (AAC CAG ATC TCG CGC GAG AAC) and *czc*R (CGG CAACAC CAG TAG GGT CAG). Polymerase chain reaction (50 μl) mixture contained 0.5 μM of each primer, 200 μM dNTPS, 1.0 U Taq DNA Polmerase, PCR buffer supplied with the enzyme and 1 μl (75 ng) of template DNA. The total volume of the reaction mixture was maintained with sterilized triple distilled water. PCR was performed in ‘BioRad iQ^TM^5 multicolor real-time PCR detection system’ and was carried out as follows: a single denaturation step at 95 °C for 5 min followed by a 36-cycle program which included denaturation at 94 °C for 1 min, annealing at 61.6 °C for 30 s and extension 72 °C for 1 min and a final extension at 72 °C for 10 min.

### Cloning and sequencing of PCR products

The purified products of *czc* gene were ligated with pDrive vector overnight at 4 °C using Qiagen cloning kit, Hilden, Germany and were transformed into *E.coli* DH5α. DNA sequencing of single pass of *czc* gene was done using primer T7. The sequence thus obtained was analyzed using BLASTn search (http://www.ncbi.nlm.nih.gov/Blast).

### Atomic absorption spectroscopy (AAS) and transmission electron microscopy (TEM)

To confirm efflux or intracellular accumulation of metal, AAS studies were done by combining the protocols given by Roane et al. 2001 and Vasudevan et al. 2001. The strains (SB32 and SB35) were grown both in the presence and absence (control) of 0.1 mM CdCl_2_ and 25 ml of sample was drawn at different intervals of time, i.e., during the lag, log, stationary and death phase. The samples were centrifuged at 7,000 rpm for 10 min at 4 °C. The supernatant was collected and passed through 0.22 μm pore size filters with the help of 10 ml disposables syringe and stored at 4 °C for further metal analysis by flame atomic absorption spectrophotometer. The pellets were dried overnight at 90 °C in the oven and weight was noted. The dried pellets (4–200 mg) were then acid lysed overnight by 5 ml concentrated HNO_3_ and incinerated on sand bath for 4–6 h at slow heating (45–50 °C). Further, 1 ml of concentrated HNO_3_ and per-chloric acid (60 %) in the ratio 6:1 was added and again kept for incineration on sand bath for 3 h till white residue is formed. The white residue was dissolved in 50 ml of deionized water. The concentration of Cd was determined by flame atomic absorption spectrophotometer at 228 nm with lamp current 3 mA.

Consequently, samples of both control and treated cells were drawn during the late log phase for TEM analysis. The strains were grown in 25 ml nutrient broth in the presence and absence of 0.1 mM CdCl_2_ and 5.0 ml sample from each experimental flask was drawn after 24 h. The samples were centrifuged at 10,000 rpm for 10 min at 4 °C. The supernatant was discarded and the pellet was washed with 5 ml PBS (pH 7.4, 0.1 M) for four times. Centrifugation was done at 8,000 rpm for 10 min at 4 °C. The pelleted cells were fixed overnight in 1 ml fixative [2.5 % glutaraldehyde and 2 % paraformaldehyde in (pH 7.4) sodium phosphate buffer] and rinsed twice with phosphate buffer for 1 h. Further, the samples were washed three times with 0.1 M phosphate buffer saline at pH 7.4 for 15 min and then fixed for 1 h at room temperature in 1 % osmium tetraoxide. The samples were dehydrated in three changes of 50 % alcohol for 15 min, four changes of absolute alcohol for 15 min and two changes of 100 % toluene for 30 min each, before being transferred to a mixture of equal parts of araldite and toluene overnight at room temperature. Impregnation was carried out in the fresh change of araldite and continued for 2 days. The samples were finally embedded in fresh araldite and polymerized for 3 days. The ultra thin sections (70–80 nm) were cut on a microtome and mounted on uncoated copper grids. The sections were stained in a saturated solution of uranyl acetate in 50 % alcohol for 15 min followed by lead citrate for 15 min and examined in 100 kV transmission electron microscope (JEOL, JEM 1011).

### In situ characterization

Seeds were sown in the pot filled with alkaline soil (pH was approximately 8.6 ± 0.2). To detect the remediation ability of strains and subsequent effect to soybean, 124 μM CdCl_2_ was added to the soil (whereas, the bacterial strains used in this study can tolerate much higher concentration, but at the concentration >124 μM, plants were unable to grow even in presence of bioinoculants). Each treatment was taken individually as indicated below:

1. Uninoculated soil (control)
2. Uninoculated soil with Cd
3. Inoculated soil with SB35
4. Inoculated with SB35 in Cd-contaminated soil
5. Inoculated soil with SB32
6. Inoculated with SB32 in Cd-contaminated soil

The inoculated pots of soybean were kept in net house at a temperature of 30 ± 5 °C. Pots were irrigated with tap water to maintain moisture content. Fifteen seeds were sown per pot, with three replicates per treatment. Plants were uprooted after 60 days of cultivation. Plant height and wet weight were measured before the plant biomass was oven dried. The agronomical parameters of plant were measured after harvesting.

### Heavy metal analysis

#### Plant materials

Plants were harvested and roots were washed extensively in several changes of a solution containing 5 mM Tris HCl (pH 6.0) and 5 mM EDTA, and then distilled water to remove non-specifically bound metal ions. Shoots and roots were oven dried separately at 60 °C for 24 h followed by 70 °C for 3 h. Aliquots (1 g) of dried leaves were ground in a porcelain mortar while dried roots were extensively minced with a razor blade and then the samples were analyzed by modified methods of wet ashing (Burd et al. 2000). Wet ashing was performed by placing aliquots of dried material in 10 ml of concentrated nitric acid at 70 °C till the brown vapor subsides. Subsequently, 10 ml of diacid mixture (HNO_3_ and HClO_4_, 3:1 ratio) was added and left till they reduced to 1 ml. Later 5 ml of 6 NHCl for 1 h was added and cooled. The digested samples were brought to 50 ml with deionized water and heavy metal analysis was done using a flame atomic absorption spectrophotometer. The instrument was zeroed with 1 % HNO_3_ blanks. Samples in triplicate were taken.

#### Soil

Soil samples were collected periodically and analyzed for residual Cd concentration. To analyze heavy metals contents in soil, 10 g of dried soil was taken in 125 ml conical flasks. Then 20 ml of diethylene triamine penta acetic acid (DTPA) extracting solution [(1 l DTPA extracting solution: dissolve 13.1 ml reagent grade triethanolamine (TEA), 1.967 g DTPA (AR grade) and add 1.47 g of CaCl_2_·2H_2_0 in 100 ml of triple distilled water, pH 7.3 ± 0.5) was shaken for 2 h at 120 cycles min^−1^ and filtrate was analyzed for Cd using flame atomic absorption spectrophotometer (Gupta 1993). Samples were taken in triplicate.

## Results and discussion

The strains SB32 and SB35 chosen for this study were tolerant to 5 mM CdCl_2_. Further, psychrotolerant strain SB32 showed optimum growth at 20 °C, while, alkalophilic strain SB35 exhibited optimum growth at pH 9.0. The partial sequencing of 16srDNA of the strains revealed the isolates to be *Pseudomonas putida* (SB32) and *Pseudomonas monteilli* (SB35) with accession numbers: HQ610451 and HQ864710, respectively.

### Molecular characterization

**Fig. 1:**
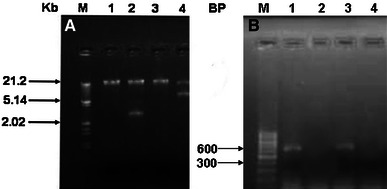
**a** Plasmid DNA profile of cadmium-resistant strains. *Lane M* λ DNA/*Eco*R1/*Hin*dIII double digest; *lanes 1–4* cadmium-resistant strains grown in the absence and presence of CdCl_2_; *1, 2* SB32; *3, 4* SB35. **b** Amplified czc gene from plasmid and genomic DNA of cadmium-resistant strains. *Lane M* 100 bp ladder; *lanes 1, 3* amplified *czc* gene from plasmid DNA of strains SB32 and SB35, respectively; *lanes 2, 4* amplified czc gene from genomic DNA of strains SB32 and SB35, respectively

Cadmium-resistant plasmid and chromosomal operons have also been reported by other workers (Lebrun et al. 1994; Horitsu et al. 1985; Lee et al. 2001; El-Deeb 2009; Mullapudi et al. 2010), therefore, to identify the location of the resistant gene; an attempt was made to amplify the *czc* gene from both plasmid and genomic DNA of the strains. The plasmid DNA profile revealed the presence of plasmid DNA of ∼5 kb (Fig. 1a). The plasmid DNA isolated from both strains, when subjected to PCR for amplification of the *czc* gene, produced an amplicon of approximate to 650 bp, whereas no amplicon was obtained in case of genomic DNA in both the strains (Fig. 1b). Also, *E.coli* DH5α which served as a negative control did not show any amplification. The presence of amplified product from the plasmid DNA of strains SB32 and SB35 indicated the presence of *czc* gene which is responsible for the efflux of three metal cations, viz. cobalt, zinc and cadmium thereby giving a clear indication of efflux mechanism. The amplicons were sequenced and when compared with the available database using BLASTn search, sequence homology of the cloned genes showed that the amplicons contained a partial *czcA* gene sequence. The nucleotide sequence of the cloned gene of strains SB32 and SB35 showed 95 % similarity with the *czcA* gene of *Stenotrophomonas maltophilia* D457 (accession number HE798556) and 94 % similarity with putative heavy metal efflux pump *czc*A family of *S. maltophilia* JV3 (accession number CP002986). Further, alignment of the translated product showed similarity with the heavy metal efflux pump, *czcA* family of *S. maltophilia* R551, *Stenotrophomonas* sp. SKA14 and *Acidovorax delafieldii* 2AN, thereby indicating the presence of an plasmid-mediated efflux mechanism of resistance in the strains. The *czc* gene sequences of strains SB32 and SB35 were deposited in gene bank database under accession numbers: KC750207 and KC750208, respectively. The mechanism was further confirmed by AAS and TEM analysis.

### Atomic absorption spectroscopy and transmission electron microscopy

**Fig. 2:**
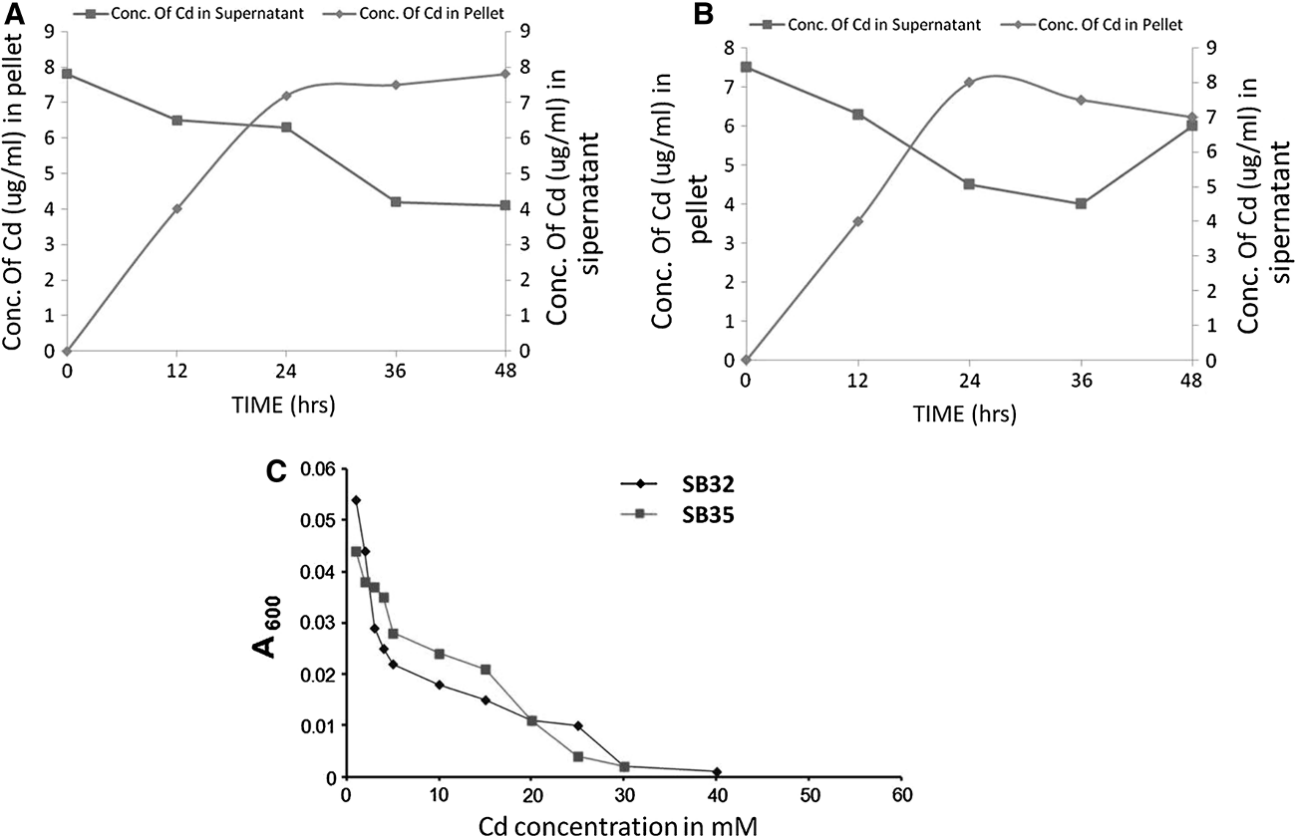
Cadmium content in pellet and supernatant fraction of **a***P. putida* strain SB32 and **b***P. monteilli* strain SB35 at different time intervals of the growth profile. **c** Correlation between growth profile and concentrations of cadmium isolates SB32 and SB35. Cells were grown in nutrient broth for 18 h

AAS analysis of strain SB32 and SB35 showed that the concentration of Cd in strain SB32, increased in the bacterial cells with the start of log phase (12 h) and reached a maximum of 7.5 μg mL^−1^ of dry weight of the cell. However, in the supernatant, the concentration of Cd followed was reversed, i.e., the Cd concentration decreased with the start of log phase (Fig. 2a).

Further, in strain SB35, the AAS results showed that the concentration of Cadmium increased with the start of log phase (12 h) and reached to a maximum of 8 μg ml^−1^ of the dry weight and then decreased after 12 h, i.e., with the end of log phase. However, in the supernatant the concentration of cadmium followed the reverse trend, i.e., the concentration decreased with the start of log phase followed by an increase in the late log phase (Fig. 2b). This clearly indicates the Cd uptake/bioremediation ability of both *Pseudomonas* strains SB32 and SB35. Furthermore, the AAS data were substantiated by TEM.

**Fig. 3:**
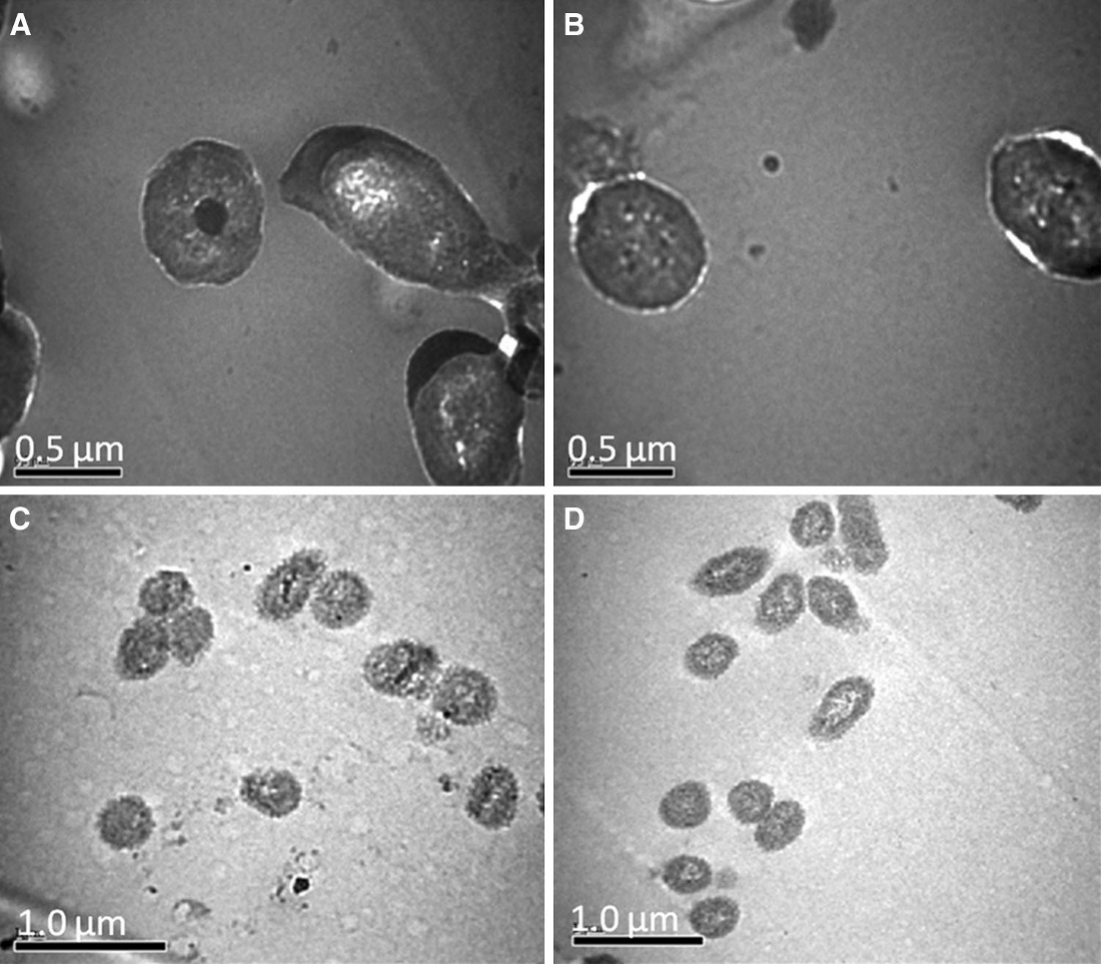
Transmission electron micrographs of **a** and **b***P. monteilli* SB35 in absence and presence of Cd, respectively, at a magnification of ×50,000; **c**, and **d***P. putida* SB32 in absence and presence of Cd, respectively at a magnification of ×40,000

TEM analysis revealed an increase in cell size of both strains in the presence of Cd (Fig. 3). Cell length was found increased by 51.13 % in SB32, while, in SB35 the increase was of 25.2 %, although there was no change in cell width. It has been reported previously that SEM analysis of *Pseudomonas aeruginosa* strain MCCB 102 showed an increase in cell size due to Cd together with lead accumulation in the cell wall and along the external cell surfaces (Zolgharnein et al. 2010). However, in this study, the cell length of strain SB32 and SB35 increased due to the entry of metal, but no further deformations were observed. This may be due to the presence of the *czc* gene which is responsible for the efflux of metal ions from the cells. The metal, which is taken into the cells by fast and unspecific transport for Mg^2+^ ions, is actively extruded by products of *czc* resistance determinants (Nies 2000). The ability of a bioinoculant to colonize aggressively makes them the preferred choice for bioremediation studies. Considerable information is available with respect to their use in natural environment but little is known about the effect of pH on bacterial survival, persistence and its subsequent effect on their bioremediation ability. So considering this point, in situ study was conducted with these diversified extremophilic Cd-resistant strains to establish a relationship between pH, temperature, their survival and subsequent effect on soybean growth.

### Impact of bioinoculants on Cd toxicity in soybean

**Fig. 4:**
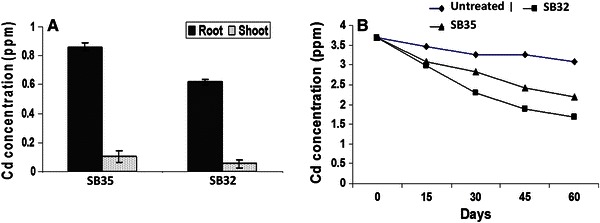
**a** Comparative cadmium accumulation in soybean in the presence of *P. monteilli SB35* and *P. putida* SB32, respectively (*n* = 3, mean ± SEM). **b** Comparative DTPA extractable residual cadmium accumulation in the presence of *P. monteilli* SB35 and *P. putida* SB32, respectively (*n* = 3, mean ± SEM)

After 60 days of germination, the plants were harvested and comparative cadmium accumulation in roots and shoots was measured. Both of the strains were compared for their ability to inhibit cadmium accumulation in plants. Cd toxicity caused a significant reduction in shoot length, root length, fresh and dry weight of soybean, and the magnitude of reduction was 5.0, 30.2, 15.45 and 25.6 % relative to control, respectively (Table 1). Nevertheless, bioinoculation of strains (SB32 and SB35) in Cd-polluted soil increased the agronomical parameters in comparison with uninoculated soil. Such comparison revealed that *P. monteilli* SB35 strain was able to reduce cadmium accumulation more than *P. putida* SB32 strain (Table 1) and reduction was found to be 47.5 and 56.9 % by *P. monteilli* SB35, than 26.9 and 17.6 % by *P. putida* SB32 in root and shoot, respectively (Fig. 4a). While comparing the effects of the *P. putida* SB32 and *P. monteilli* SB35 on the Cd content reduction in soil (Table 1), it appeared that the strain SB35 was more effective in reducing Cd concentration than SB32 and it reduces 1.33 times more than latter in alkaline soil type (Fig. 4b). As evident from the data (Table 1), in alkaline soil, *P. monteilli* SB35 was found to be more efficient in enhancing plant growth than *P. putida* SB32 strain. Cadmium mainly occurs as the free metal ion Cd^2+^ (Bingham and Page 1975) and ion exchange mechanisms have a dominating influence on metal concentration in soil solution. Changes in pH exert both a biological and chemical effect on metal ion toxicity (Campbell and Stokes 1985). Low pH favors greater metal ion solubility, and, in the absence of complexing ions, reduced speciation of the metal ion, which tends to increase toxicity compared to higher pH. However, low pH also enhances competition between H^+^ and metal ion for cell surface binding sites, which tends to decrease metal ion toxicity.

**Table 1:** Two way ANOVA depicting the effect of *P. putida* SB32 and *P. monteilli* SB35 on Soybean under greenhouse conditions in alkaline soil (30 ± 5 °C) after 60 days of germination, respectively

	Root length^a^ (cm)	Shoot length^a^ (cm)	Wet weight^a^ (g)	Dry weight^a^ (g)	Chlorophyll^b^ (mg g^−1^)
In absence of cadmium					
Mean (control)	11.4 ± 0.11^d^	33.7 ± 0.18	4.16 ± 0.17	2.41 ± 0.63	3.1 ± 0.17
Mean (SB35 strain treated)	12.9 ± 0.85 (13.1 %)^e^	39.7 ± 0.16 (17.8 %)	4.83 ± 0.14 (16.1 %)	3.07 ± 0.11 (6.0 %)	3.3 ± 0.12 (3.5 %)
Mean (SB32 strain treated)	12.5 ± 0.61 (9.6 %)	37.2 ± 0.24 (10.3 %)	2.78 ± 0.10 (46.9 %)	2.96 ± 0.70 (45.4 %)	3.05 ± 0.18 (7.1 %)
In presence of cadmium					
Mean (control)	8.32 ± 0.45	28.1 ± 0.16	2.23 ± 0.18	1.34 ± 0.87	1.7 ± 0.15
Mean (SB35 strain treated)	10.5 ± 0.55 (26.2 %)	31.4 ± 0.17 (11.7 %)	3.5 ± 0.11 (56.9 %)	2.12 ± 0.95 (58.2 %)	2.5 ± 0.21 (47 %)
Mean (SB32 strain treated)	12.1 ± 0.27 (45.4 %)	29.7 ± 0.18 (5.6 %)	2.47 ± 0.88 (10.7 %)	1.66 ± 0.92 (23.8 %)	1.97 ± 0.33 (15.8 %)
Critical difference at 5 %					
	2.22	4.66	0.44	0.26	0.41

^a^Mean of ten replicates

^b^Mean of ten replicates

^c^Mean of four replicates

^d^The reported values are ±SEM

^e^Values indicate the % increase over the respective control

The increase in agronomical parameters in the presence of cadmium in the soil documented the growth promotory as well as bioremediation potential of bioinoculants. Similar observations were made earlier by Burd et al. (2000). However, toxicity of these metals depends on the soil pH. Further, cadmium content analysis revealed alkalophilic *P. monteilli* SB35 was more effective than *P. putida* SB32 strain. Both of the strains were able to reduce the cadmium accumulation in plants and soil significantly. This shows the importance of abiotic factor such as pH for survival and growth of bioinoculants for the establishment of a threshold population of viable inoculant which is an important prerequisite for the successful bioremediation strategy.

## Conclusion

It is clear from the above results that resistance to Cd in both the diversified extremophilic strains SB32 and SB35 of *Pseudomonas* is due to the *czc* gene present on the plasmid DNA and involves metal binding and/or an efflux mechanism of resistance. Furthermore, the results of in situ trial, AAS, TEM and EDAX analysis suggest that the strains may have considerable potential as an agent for bioremediation under natural conditions with reference to Cd. Moreover, knowledge of the gene employed and the mechanism involved in Cd resistance may be used to design a tailor-made plant growth promotory bioinoculant.
